# Aldosterone in Chronic Kidney Disease

**DOI:** 10.3390/biomedicines13092081

**Published:** 2025-08-26

**Authors:** Paula Polenus, Ana Đuran, Sandra Karanović Štambuk

**Affiliations:** 1 School of Medicine, University of Zagreb, 10000 Zagreb, Croatia; 2 Department of Pulmonology, University Hospital Center Split, 21000 Split, Croatia; ana.djuran@gmail.com; 3 Division of Nephrology, Arterial Hypertension, Dialysis, and Transplantation, Department of Internal Medicine, University Hospital Center Zagreb, 10000 Zagreb, Croatia

**Keywords:** aldosterone, aldosterone synthase inhibitors, chronic kidney disease, mineralocorticoid receptors, non-steroidal mineralocorticoid receptor antagonists

## Abstract

Chronic kidney disease (CKD) is a global health challenge, marked by significant morbidity and mortality and a rising economic burden. Despite established therapies such as renin–angiotensin system (RAS) inhibitors and SGLT2 inhibitors, a substantial residual risk of CKD progression and cardiovascular events persists. This gap is largely attributed to the sustained overactivation of the mineralocorticoid receptors by aldosterone, a key driver of renal inflammation and fibrosis. This review aims to bridge the understanding between aldosterone’s intricate pathophysiology and emerging therapeutic strategies designed to address this unmet clinical need. We discuss the physiological regulation of aldosterone synthesis and secretion, the phenomenon of aldosterone breakthrough under conventional RAS blockade and the diverse mechanisms through which aldosterone mediates kidney damage. We evaluate novel non-steroidal mineralocorticoid receptor antagonists, exemplified by finerenone, which demonstrate superior safety profiles and valid efficacy in reducing renal and cardiovascular outcomes in clinical trials. Additionally, we examine aldosterone synthase inhibitors as an upstream therapeutic approach to directly reduce aldosterone production. These novel agents represent promising avenues to mitigate residual risk and improve long-term outcomes for patients with CKD.

## 1. Introduction

Kidney diseases are unquestionably among the most common health conditions worldwide, affecting over 850 million people globally—of which the vast majority (over 840 million) are cases of chronic kidney disease (CKD) [1]. Since the year 2000, when it ranked as the 19th leading global cause of death, CKD has risen to 9th place, with the number of deaths increasing by 95% to 1.53 million in 2021 [2]. CKD is projected to become the fifth leading cause of mortality globally by 2040 [3]. In addition, economic repercussions of CKD are extensive, placing an increasing burden on individuals, healthcare systems, and national economies worldwide. For example, many developed countries allocate 2–3% of their annual healthcare budgets to the management of end-stage kidney disease (ESKD), despite this population comprising only about 0.03% of the total [4].

Numerous external factors contribute to developing CKD, primarily through hemodynamic and metabolic overload of individual nephrons. These contributing factors include both non-modifiable (e.g., genetic predisposition) and modifiable (e.g., diabetes and hypertension) risk factors [5]. The progression of CKD, characterized by gradual nephron loss, is associated with both local renal inflammation and low-grade systemic inflammation [6]. Key mechanisms underlying this chronic inflammatory state include the accumulation of uremic toxins, oxidative stress, and the activation of the local renin–angiotensin–aldosterone system (RAAS), particularly through the stimulation of the mineralocorticoid receptors (MR) [7,8,9]. This inflammatory environment promotes tissue remodeling and fibrosis through a process analogous to wound healing. Histologically, this manifests as glomerulosclerosis, tubular atrophy, interstitial fibrosis, and vascular sclerosis.

Persistent local activation of the RAAS—via angiotensin II (Ang II) and aldosterone-mediated MR activation—is considered a major driver of fibrotic changes in the kidney [9]. Among other mediators of this process is endothelin, a potent vasoconstrictor. Endothelin contributes to the development of fibrosis by binding to its type A and type B receptors [10]. Blocking endothelin receptors is one of the therapeutic avenues actively being investigated in clinical settings [11,12].

The foundational pharmacological approach involving the use of ACE inhibitors (ACEis) and angiotensin receptor blockers (ARBs) has demonstrated efficacy in reducing proteinuria and delaying the progression of CKD. This is attributed to the significant role of Ang II, a key component of the RAAS, in promoting fibrosis and the excessive proliferation of mesangial cells and atrophy of tubular cells, which ultimately leads to nephron loss and a decline in renal function. However, it is well-established that blocking the RAAS with ACEis and ARBs alone is often insufficient to halt CKD progression, somewhat due to other mechanisms involved and somewhat due to the ACEi/ARB bypass effect, as well as the phenomenon of aldosterone breakthrough [13]. Therefore, despite ACE/ARB inhibition and the introduction of sodium–glucose co-transporter 2 (SGLT2) inhibitors, a significant residual risk remains and in a subset of patients, renal function continues to decline, ultimately leading to ESKD [14].

Mineralocorticoid receptor antagonists (MRAs), the oldest of which—spironolactone—has been known for over 60 years [15], are associated with a significant reduction in the risk of cardiovascular events [16,17], and effective treatment of heart failure [18] and resistant hypertension [19], as well as albuminuria reduction, both in diabetic and non-diabetic CKD [20]. Despite robust evidence supporting their efficacy, concerns regarding hyperkalemia and other adverse effects have led to their underutilization in key patient populations [21] but also limited conduction of randomized clinical trials with hard kidney endpoints such as progression to dialysis. These limitations underscore the need for newer therapeutic options that offer a more favorable safety profile while maintaining comparable clinical benefits.

This review aims to explore the role of aldosterone in CKD without discussing its cardiovascular implications, and to evaluate the clinical utility of its pathway as a therapeutic target in light of existing and new treatment options.

## 2. Aldosterone Synthesis and Secretion

Aldosterone is a steroid hormone classified as a mineralocorticoid, primarily synthesized in the zona glomerulosa of the adrenal cortex; however, evidence also suggests that extra-adrenal aldosterone production occurs in the brain, heart, blood vessels, adipose tissue, and the kidney [22]. The synthesis of aldosterone involves a series of enzymatic reactions, with cytochrome P450 11β2 (CYP11β2) also named aldosterone synthase, converting deoxycorticosterone into aldosterone in the last step. Once released, aldosterone travels to the kidneys, where it exerts its genomic effects, acting on the late distal tubule and collecting duct of nephrons to promote the reabsorption of sodium and, consequently, water into the bloodstream, while increasing the excretion of potassium into the urine. This action leads to an increase in blood volume and, subsequently, blood pressure, which, through negative feedback, suppresses further activation of the RAAS.

The secretion of aldosterone is controlled by several factors, with the Ang II and plasma potassium levels being the most influential. Ang II stimulates aldosterone secretion by binding to angiotensin II type 1 receptors on glomerulosa cells, resulting in increased intracellular calcium levels and the activation of aldosterone synthase CYP11β2; additionally, in vitro studies show that angiotensin III might also have some effect on aldosterone secretion by binding to type 2 receptors [23]. Elevated extracellular potassium concentrations directly stimulate aldosterone secretion by depolarizing glomerulosa cells and triggering an influx of calcium. Adrenocorticotropic hormone, released by the anterior pituitary gland, binds to its receptor on glomerulosa cells, activating the cAMP/PKA pathway and promoting steroidogenesis, including the synthesis of aldosterone. Notably, research suggests the existence of a local paracrine/autocrine negative feedback loop within the zona glomerulosa, mediated by the mineralocorticoid receptor itself [24]. Aldosterone-producing cells of the adrenal gland are also subject to paracrine regulation by substances such as leptin, endothelin, and substance P with its direct and mast-cell-mediated indirect influence on aldosterone secretion [25].

The regulation of aldosterone secretion remains to some point incompletely understood, which partly explains why the phenomenon of “aldosterone breakthrough” continues to be an active area of research. This phenomenon refers to the re-elevation of aldosterone levels following an initial suppression achieved through monotherapy with ACEis or ARBs. Notably, aldosterone breakthrough has been observed in approximately 30–40% of patients undergoing such treatments [26], a significant finding given that its occurrence during renin–angiotensin system (RAS) blockade—particularly in the context of diabetic nephropathy—is associated with an accelerated decline in GFR [27]. Mechanistically, aldosterone breakthrough may result from several factors, including the following: ACE-independent pathways such as angiotensin II producing chymase, physiological stimuli (sodium and potassium levels and circulating volume) that can potentially override RAS inhibitors, or pharmacokinetic factors that influence the effectiveness of RAS inhibitors over time [23,28].

## 3. Mineralocorticoid Receptor (Over)Activation

Aldosterone is considered the primary ligand for MRs, which are expressed in various tissues and cell types beyond the epithelial cells of the distal tubule, colon, and the sweat glands, including the central nervous system, adipocytes, cardiomyocytes, fibroblasts, podocytes, mesangial cells, inflammatory cells (T cells and myeloid cells), endothelial cells, and (vascular) smooth muscle cells [29,30].

However, in addition to aldosterone, MRs can be also activated by cortisol as well as by nonligand activation due to Ras-related C3 botulinum toxin substrate 1 (Rac-1), oxidative stress, elevated glucose, and high salt levels [31]. Namely, MR exhibits similar binding affinities for both aldosterone and cortisol. Thus, cortisol could lead to non-selective MR activation in the absence of protective mechanisms. The enzyme 11β-hydroxysteroid dehydrogenase type 2 (11β-HSD2) functions as a high-affinity dehydrogenase, rapidly converting biologically active glucocorticoids (cortisol in humans) into their inactive 11-keto forms (cortisone), with its primary site of action being epithelial cells of the renal distal tubules and collecting ducts, but also present in the renal endothelium, colon, brain nuclei, and vessel walls [32]. This conversion renders tissues possessing both the enzyme 11β-HSD2 and the MR selectively responsive to aldosterone. In CKD, alterations in the expression or activity of 11β-HSD2 may disrupt the local balance between cortisol- and aldosterone-mediated MR activation [30]. Cortisol’s ability to activate the MR is particularly relevant in extra-renal tissues and in specific renal cell types with low 11β-HSD2 expression such as podocytes or fibroblasts [9]. Additionally, hyperglycemia, high salt intake, and mechanical stress promote Rac1 activation, which in turn activates MR independently of aldosterone or cortisol. All of this suggests that MR activation in CKD may not be driven exclusively by aldosterone. Depending on the tissue type and the local enzymatic milieu, cortisol and other factors may significantly contribute to MR overactivation in CKD [31].

Classically, upon aldosterone binding, the MR undergoes conformational changes, disassociates from chaperone proteins, and translocates to the nucleus where it acts as a ligand-dependent transcription factor. Within the nucleus, MR binds to specific hormone response elements in target gene promoters, thereby modulating gene expression. This genomic modulation influences key cellular processes, including the regulation of epithelial sodium channels (ENaC) in the kidney via downstream kinases like circulating glucocorticoid-regulated kinase 1 [33], facilitating sodium–water reabsorption and potassium secretion. This is known as aldosterone’s genomic pathway.

In a state of disease, aldosterone triggers several pathologic processes including inflammation and fibrosis through the genomic pathway, but also through a rapid, non-genomic one [34]. Namely, beyond the classical genomic effects, aldosterone also triggers rapid, non-genomic responses, primarily through the G protein-coupled estrogen receptor GPER/GPR30, though the membrane-associated MR may also contribute [34]. These effects involve swift changes in intracellular ion concentrations, such as calcium, and the activation of signaling cascades, such as c-Src, leading to the transactivation of growth factor receptors (e.g., epidermal growth factor receptor) [35] and subsequent activation of kinases such as mitogen-activated protein kinase and protein kinase C. For instance, in vascular smooth muscle cells, aldosterone-induced NOX2/NOX4 activation generates reactive oxygen species (ROS) [36]. Such pathways contribute to cellular proliferation, inflammation, and fibrosis, particularly in vascular and renal tissues. These non-genomic pathways often operate in concert with other signaling systems, potentially involving crosstalk between MR and angiotensin II type 1 receptor pathways [37], further contributing to target organ damage.

The potential for aldosterone to act through receptors other than the classical MR—via distinct signaling pathways—has important implications for therapeutic strategies targeting aldosterone in CKD. If some of aldosterone’s detrimental effects are mediated by non-classical receptors, then the selective blockade of classical MRs may be insufficient. In particular, non-classical MRs are not inhibited by spironolactone [38], and current research on non-steroidal MRAs has yet to provide a definitive answer regarding their efficacy against non-genomic MR signaling; however, aldosterone synthase inhibitors (ASI) may be helpful in this context.

## 4. Aldosterone-Induced Kidney Damage

The kidney-damaging effects of aldosterone have been recognized for nearly as long as the hormone itself has been known [39]. Today, it is well established that elevated aldosterone levels in individuals with CKD are independently associated with an increased risk of CKD progression promoting glomerular injury, interstitial inflammation, and progressive fibrosis [25].

Animal studies, dating back to the late 1990s and 2000s, demonstrated that the targeted blockade of aldosterone mitigated glomerulosclerosis and reduced urinary protein levels, whereas the co-infusion of aldosterone reinstated damage despite concurrent ACEi and ARB administration [40]. These findings solidified aldosterone’s direct involvement in kidney pathology, particularly through the stimulation of inflammatory responses and fibrosis [41].

MR activation contributes to CKD progression not only through hemodynamic alterations—increased intraglomerular pressure and potential ischemia—but also through non-hemodynamic mechanisms. The activation of MRs is generally associated with vasoconstriction and increased blood pressure, an effect mediated by several signaling pathways. Vasoconstriction is promoted through mediators such as endothelin-1 and the activation of the angiotensin II type 1 receptor. Additionally, MR activation impairs vasodilation by reducing signaling through the endothelin B receptor, decreasing endothelial nitric oxide synthase activation and downregulating angiotensin II type 2 receptor activity [42].

Beyond hemodynamic effects, MR overactivation promotes inflammation, oxidative stress, and fibrosis in multiple tissues, including the kidney. These effects are mediated through direct actions on renal cells (e.g., podocytes, mesangial cells, endothelial cells, and tubular epithelial cells) as well as immune cells (e.g., myeloid cells such as macrophages). Compelling evidence supporting these effects has been obtained from extensive research on chronic MR activation, using different approaches such as in vitro studies, animal models (including MR knockout models), and the application of MRAs [43].

Aldosterone plays a direct role in promoting inflammation by upregulating the expression of key inflammatory and adhesion molecules such as intercellular adhesion molecule 1, vascular cell adhesion molecule 1, cyclooxygenase 2, monocyte chemotactic protein 1, and osteopontin prior to inflammatory cell infiltration [44]. This inflammatory environment, coupled with increased nicotinamide adenine dinucleotide phosphate oxidase activity (especially in podocytes and mesangial cells)—a major source of ROS in renal, cardiac, and vascular tissues—contributes significantly to oxidative stress and tissue injury [45]. Furthermore, MR activation drives pro-fibrotic processes; for instance, the binding of aldosterone to the MR activates signaling pathways such as the epidermal growth factor receptor and PI3K/MAPK cascades, which promotes fibroblast proliferation [46] and ultimately leads to fibrosis.

Neutrophil Gelatinase-Associated Lipocalin (NGAL), a small circulating protein and early biomarker of renal injury, is increasingly recognized for its role in kidney pathology. NGAL is an MR target in the cardiovascular system that contributes to aldosterone-MR-mediated pro-fibrotic effects through the induction of collagen-I, galectin-3 (Gal-3), and cardiotrophin-1 [47]. Studies in CKD models have shown NGAL’s causative role in kidney damage as well, with NGAL deficiency reducing renal lesions, tubulointerstitial fibrosis, and tubular cell death, while exogenous NGAL exacerbates injury [48]. Similarly, Gal-3, a β-galactoside-binding lectin, is another crucial mediator implicated in inflammation and fibrosis. Elevated Gal-3 levels are associated with CKD progression, and Gal-3 is thought to mediate the profibrotic effects of aldosterone and the MR in the kidney, including promoting macrophage infiltration and myofibroblast activation [49].

In myeloid cells, such as macrophages, MR activation leads to increased interleukin-4 receptor signaling, thereby promoting their differentiation into a pro-inflammatory (M1) phenotype. This contributes to the inflammatory milieu within the kidney and exacerbates renal injury. Aldosterone has also been shown to promote renal inflammatory responses by upregulating NF-κB expression [24], crucial in mediating macrophage-induced injury. MR inhibition in macrophages has been shown to reduce pro-inflammatory markers and promote an anti-inflammatory (M2) phenotype, which may offer protection against the transition from acute kidney injury to CKD [50,51].

MR activation in various cell types including those in the kidney, also induces the expression of pro-fibrotic factors such as connective tissue growth factor, transforming growth factor-beta (TGF-β), and plasminogen activator inhibitor-1 (PAI-1), contributing to progressive kidney injury and fibrosis [52]. Additionally, renal podocytes have been found to activate the nucleotide-binding oligomerization domain–like receptor family pyrin domain-containing 3 inflammasome in response to aldosterone, further promoting the development of injury and consequent proteinuria and CKD progression [53].

## 5. Therapeutic Targeting of the Aldosterone Pathway in CKD

The comprehensive understanding of aldosterone’s multifaceted role in promoting kidney damage, through both hemodynamic and a complex array of inflammatory and fibrotic mechanisms, underscores the need for targeted therapeutic strategies in CKD. Established therapies like RAS inhibitors and SGLT2 inhibitors offer significant renoprotective and cardioprotective effects; however, they do not sufficiently address the profound MR-mediated as well as MR-independent fibrotic and inflammatory pathways [54,55,56]. As a result, patients often face a significant residual risk of CKD progression and cardiovascular events [57].

To address this therapeutic gap, two primary strategies have emerged to directly counteract the pathological effects of aldosterone. The first involves the development of MRAs, which directly block the downstream effects of aldosterone at the receptor level [58]. The second, a novel and potentially complementary approach, focuses on aldosterone synthase inhibitors, ASIs. These agents act upstream by reducing aldosterone production itself, thereby potentially mitigating both MR-dependent and MR-independent effects of aldosterone and offering a more complete blockade of the pathway [59]. The remainder of this review will be dedicated to these emerging therapeutic approaches.

### 5.1. Steroidal MR Antagonists

Following the discovery of aldosterone, numerous synthetic steroids were investigated for their potential to inhibit its sodium-retaining and potassium-excreting effects. In the 1950s, spironolactone was introduced as the first steroidal MRA. It was sold under the brand name “Aldactone” and it initially launched as a diuretic and natriuretic drug. At first it was used as a mainstay treatment for primary aldosteronism and for other clinical entities, such as idiopathic edema, liver cirrhosis, and hypertension [60].

While spironolactone has been well-established in the management of heart failure, increasing evidence supports its utility in CKD as well [17,61]. Spironolactone is frequently employed as an adjunct therapy alongside ACEis or ARBs, which remain cornerstone treatments for CKD. When used in combination, spironolactone and RAS inhibitors have demonstrated additive effects in reducing proteinuria, a key marker of kidney damage and a surrogate marker for CKD progression, thereby helping to slow the disease advancement [61,62]. Furthermore, spironolactone is classified as a prodrug with a relatively short half-life of less than two hours. Nonetheless, its pharmacodynamic effects are primarily mediated by its active metabolites, which possess significantly longer half-lives ranging from approximately 12 to 24 h [15]. Spironolactone competes with androgens like testosterone and dihydrotestosterone at the androgen receptor which leads to antiandrogenic side-effects, most commonly gynecomastia and impotence in males and can also induce menstrual irregularities in women [63]. To address the limitations of earlier MRAs, eplerenone, a more selective steroidal mineralocorticoid receptor antagonist, was developed and approved in the early 2000s. Compared to spironolactone, eplerenone exhibits improved receptor selectivity and a more favorable side-effect profile, particularly with respect to reduced off-target hormonal effects, but has a lower potency to MRs [64]. Importantly, studies have also shown that eplerenone can provide renoprotective benefits in patients with CKD, including significant reductions in albuminuria [65,66]. However, despite these advantages, both spironolactone and eplerenone remain associated with a risk of hyperkalemia, especially in individuals with compromised renal function [67,68].

### 5.2. Non-Steroidal MR Antagonists

The clinical use of steroidal MRAs such as spironolactone and eplerenone in CKD has been constrained by a high risk of hyperkalemia and endocrine-related adverse effects, which limit their long-term tolerability and widespread adoption. In response to these limitations, the development of non-steroidal MRAs, most notably finerenone, represents a significant therapeutic advancement [69]. With greater MR selectivity, a more favorable safety profile, and demonstrated efficacy in reducing both renal and cardiovascular outcomes in patients with diabetic kidney disease, finerenone offers a promising alternative [70]. Moreover, its targeted mechanism of action also suggests potential benefits in non-diabetic CKD [71]. Apart from finerenone, several other non-steroidal MRAs with enhanced potency and selectivity have recently undergone or are currently undergoing preclinical evaluation and clinical trials.

- Ocedurenone or KBP-5074

Ocedurenone is a novel, high-affinity, and selective non-steroidal MRA primarily tested for the treatment of arterial hypertension. In preclinical studies, it demonstrated dose-dependent reductions in blood pressure and urinary albumin excretion in various hypertensive rat models, including the Dahl salt-sensitive and stroke-prone spontaneously hypertensive rats [72]. The multicenter, randomized, double-blind, placebo-controlled BLOCK-CKD study assessed the safety and efficacy of ocedurenone in patients with resistant or inadequately controlled hypertension and advanced CKD stages (3b/4). By the end of the study, the drug achieved a statistically significant reduction in systolic blood pressure compared to placebo. Additionally, while there was a favorable trend toward a reduction in urinary albumin-to-creatinine ratio (UACR) in the ocedurenone group, this change did not reach statistical significance within the study period [73]. Although the BLOCK-CKD study demonstrated the potential of ocedurenone in managing blood pressure in patients with advanced CKD, the subsequent phase 3 CLARION-CKD trial on the treatment of uncontrolled hypertension and advanced chronic kidney disease was discontinued due to a lack of sufficient efficacy [74].

- Esaxerenone or CS-3150

CS-3150 or esaxerenone is a high-affinity and highly selective MRA, with a more than 1000-fold higher selectivity for the MR compared with the glucocorticoid, progesterone, or androgen receptor [75]. Regulatory approval of esaxerenone in Japan was primarily based on the results of the ESAX-HTN phase 3 clinical trial, which provided robust evidence of its antihypertensive efficacy and favorable safety profile in comparison with eplerenone in essential hypertensives [76]. Furthermore, the ESAX-DN trial was a pivotal phase 3 clinical study also conducted in Japan to assess the efficacy and safety of esaxerenone in patients with type 2 diabetes and moderately increased albuminuria. The trial demonstrated that the addition of esaxerenone to standard RAS inhibitor therapy significantly increased UACR remission rates and reduced the risk of progression to overt albuminuria. These results highlight esaxerenone’s potential as a valuable therapeutic option for the management of diabetic kidney disease [77].

- Balcinerenone or AZD9977

AZD9977, also known as balcinerenone, is also a non-steroidal MRA which was under investigation for its potential therapeutic benefits in patients with CKD, heart failure, and other conditions related to renal and cardiovascular disorders. The MIRACLE trial was a phase 2b, randomized, double-blind, active-controlled, multicenter clinical study and its primary objective was to evaluate the efficacy, safety, and tolerability of balcinerenone in combination with dapagliflozin in patients with heart failure and CKD. The trial aimed to determine whether this combination therapy could provide additional benefits in managing these conditions, particularly in reducing UACR, a marker of kidney damage. The trial enrolled 153 participants aged 21 years and older, with an estimated glomerular filtration rate (eGFR) between 30 and 60 mL/min/1.73 m^2^. The results of the trial showed that the addition of balcinerenone to dapagliflozin did not lead to a significantly greater reduction in UACR compared to dapagliflozin alone. In terms of safety, the trial found a dose-dependent increase in serum potassium levels and a reduction in eGFR, particularly in the group receiving the highest dose of balcinerenone [78]. While the MIRACLE trial did not demonstrate a significant benefit of adding balcinerenone to dapagliflozin in reducing UACR, it provided valuable insights into the safety profile of this combination therapy. The MIRO-CKD trial, that was recently completed, was designed to evaluate the efficacy, safety, and dose-response of balcinerenone in combination with dapagliflozin in patients with CKD and albuminuria. The primary objective was to identify an optimal dose of balcinerenone/dapagliflozin for a future phase 3 study in patients with CKD [79]. The results of the study have not yet been published. Whereas both trials aimed to assess the combination of balcinerenone and dapagliflozin in CKD patients, the MIRACLE trial focused on those with heart failure, whereas the MIRO-CKD trial included a broader CKD population.

- Apararenone or MT-3995

Apararenone is a long-acting, non-steroidal MRA. It has an exceptionally prolonged plasma half-life of 275–285 h, and its active metabolite, 1118174, demonstrates an even longer half-life ranging from 1126 to 1250 h after both single and multiple doses, due to enterohepatic recycling [80]. Apararenone was evaluated in a randomized, double-blind phase 2 study involving patients with stage 2 diabetic nephropathy in Japan. The study demonstrated a dose-dependent reduction in UACR, indicating its potential efficacy in slowing the progression of diabetic kidney disease. Over the 52-week treatment period, apararenone was well tolerated, with no clinically significant adverse effects on renal function or serum potassium levels, supporting its favorable safety profile in this patient population [81]. However, in 2021 the Mitsubishi Tanabe communicated that the development of apararenone had been discontinued.

#### Finerenone

Finerenone is a next-generation, non-steroidal MRA developed to overcome the limitations of earlier steroidal MRAs. With greater selectivity and an improved safety profile, it has demonstrated significant renal and cardiovascular benefits, particularly in patients with CKD and type 2 diabetes.

- Pharmacokinetics

Finerenone is a nonsteroidal MR antagonist distinguished by its high selectivity and strong binding affinity for the MR. It is more potent than eplerenone and at least as potent as spironolactone in terms of MR inhibition. Unlike first-generation agents such as spironolactone, finerenone exhibits minimal affinity for other steroid hormone receptors, significantly reducing the risk of off-target hormonal side effects [82]. Following oral administration in humans, finerenone is rapidly and completely absorbed, with peak plasma concentrations typically achieved within 0.5 to 1.25 h in the fasted state. Its absolute bioavailability is approximately 43.5%, limited by first-pass metabolism in both the gut wall and liver. Finerenone is primarily metabolized by CYP3A4 (approximately 90%) and to a lesser extent by CYP2C8 (10%). Elimination from plasma is rapid, with a half-life of approximately 2 to 3 h [81]. Preclinical studies in rats using carbon-14-labeled finerenone demonstrated no evidence of irreversible tissue binding or retention, with only minimal radioactivity observed in the brain. Quantitative whole-body autoradiography further indicated a balanced distribution of finerenone between cardiac and renal tissues in healthy animals [68,83].

- Mechanism of action

Finerenone mediates its therapeutic effects by blocking the MR, thereby attenuating the pathogenic actions of aldosterone in target tissues. Its nonsteroidal dihydropyridine-derived structure exhibits high affinity and selective binding to the MR, inducing a distinct bulky antagonistic conformation that forms less stable MR–ligand complexes compared to those of steroidal MRAs. This conformational instability disrupts the recruitment of key nuclear co-regulators, including steroid receptor coactivator-1, ultimately suppressing the transcription of pro-inflammatory and pro-fibrotic genes [68]. Unlike certain steroidal MRAs, finerenone lacks partial agonist activity, enabling consistent and complete MR blockade. Additionally, it impedes aldosterone-dependent nuclear translocation of the MR, a pivotal step in the activation of fibrotic and inflammatory pathways [84]. In preclinical studies, finerenone has demonstrated significant efficacy in protecting against renal damage in various CKD models. MR overactivation increases ROS production, contributing to renal dysfunction, but finerenone reduces oxidative stress, as shown by decreased markers like malondialdehyde and 8-hydroxyguanosine in animal models. It also mitigates renal inflammation, evidenced by reductions in pro-inflammatory markers such as tumor necrosis factor alpha and Monocyte Chemoattractant Protein-1, and by promoting macrophage polarization toward an anti-inflammatory M2 phenotype. In terms of renal fibrosis, finerenone has been shown to reduce the expression of pro-fibrotic markers, including PAI-1 and TGF-β, and to limit collagen deposition in various experimental CKD settings [85].

- Completed randomized clinical trials regarding finerenone use in CKD

The ARTS study was the first randomized clinical study to evaluate finerenone (BAY 94-8862) in patients with heart failure with reduced ejection fraction (HFrEF) and mild to moderate CKD, utilizing a double-blind placebo arm and an open-label spironolactone comparator. The study demonstrated that finerenone was well tolerated, with a significantly lower incidence of hyperkalemia compared to the spironolactone group. Moreover, finerenone significantly reduced N-terminal pro B-type natriuretic peptide (NT-proBNP) levels and was associated with smaller declines in eGFR and a lower incidence of worsening renal function, suggesting a more favorable renal safety profile relative to spironolactone in this patient population [86].

The ARTS-HF study compared finerenone with eplerenone in a distinct and high-risk patient population. Among patients with worsening HFrEF requiring hospitalization and who also had diabetes and/or CKD, finerenone was found to reduce NT-proBNP levels to a similar degree as eplerenone, while offering a more favorable safety profile, particularly regarding hyperkalemia [87]. The ARTS-DN trial demonstrated that adding finerenone to an ACEi or ARB significantly reduced UACR in a dose-dependent manner, with reductions ranging from 21% to 38% at doses of 7.5 to 20 mg/day compared to placebo [88]. The abovementioned ARTS-HF and ARTS-DN phase 2 trials provided essential data on the efficacy and safety of finerenone, which were instrumental in shaping the larger phase 3 studies that further confirmed the drug’s benefits in chronic kidney disease and the diabetic population. These two finerenones’ phase 3 pivotal trials, FIDELIO-DKD and FIGARO-DKD, form the foundation of the clinical evidence supporting their use in patients with chronic kidney disease associated with type 2 diabetes. The FIDELIO-DKD trial randomly assigned 5734 patients with CKD and type 2 diabetes to receive finerenone or placebo on top of the RAS blockade. Eligible patients had a UACR of 30 to less than 300 mg/g, eGFR of 25 to less than 60 mL/min/1.73 m^2^, and diabetic retinopathy, or they had a UACR of 300 to 5000 mg/g and an eGFR of 25 to less than 75 mL/min/1.73 m^2^. The primary composite outcome was kidney failure, a sustained decrease of at least 40% in the eGFR from baseline, or death from renal causes, while the secondary composite outcome included death from cardiovascular causes, nonfatal myocardial infarction, nonfatal stroke, or hospitalization for heart failure. The results of the FIDELIO-DKD study demonstrated that finerenone significantly reduces the risk of kidney disease progression and cardiovascular events when added to standard care, i.e. RAS blockade [70]. Unlike previous attempts of dual RAAS inhibition, finerenone achieved these benefits with a favorable safety profile [70]. In the FIGARO-DKD trial, finerenone therapy significantly improved cardiovascular outcomes compared with placebo in patients with type 2 diabetes and chronic kidney disease, specifically those with stage 2 to 4 CKD and moderately increased albuminuria, or stage 1 or 2 CKD with severely increased albuminuria [89]. Together, these trials demonstrated significant reductions in both kidney disease progression and cardiovascular events, findings that were further supported by the pooled FIDELITY analysis [90]. These results led to the approval of finerenone by the FDA and the EMA in 2021/22 for the treatment of patients with chronic kidney disease associated with type 2 diabetes [91]. Additionally, the FINE-HEART pooled analysis, which included the two abovementioned trials as well as data from FINEARTS-HF dealing with heart failure patients, confirmed finerenone’s efficacy on all-cause mortality, cardiovascular events, and kidney outcomes in patients with overlapping cardiovascular and kidney metabolic diseases [92].

Finally, the recently published CONFIDENCE trial, a phase 2 clinical trial evaluating adults with CKD and type 2 diabetes, was designed to test the hypothesis that early combination therapy with finerenone and empagliflozin was more effective in reducing UACR over six months than either treatment alone. The results proved the hypothesis—namely, the reduction in the UACR with combination therapy was 29% greater than that with finerenone alone and 32% greater than that with empagliflozin alone [93].

- Ongoing finerenone trial

The FIND-CKD trial represents a promising advancement in the management of CKD, particularly for patients without diabetes, a population for whom treatment options remain limited. Building on the success of finerenone in improving kidney and cardiovascular outcomes in diabetic CKD, this landmark phase 3 trial explores its potential benefits in a broader, non-diabetic CKD population. As the first study of its kind, FIND-CKD evaluates whether finerenone can slow kidney function decline and reduce cardiorenal complications in non-diabetic CKD patients. The randomized, double-blind, placebo-controlled trial enrolled 1584 adults across 24 countries, most commonly with chronic glomerulonephritis or hypertensive/ischemic nephropathy. The primary outcome is the annual rate of eGFR decline over 32 months, with secondary outcomes assessing major kidney and cardiovascular events. By targeting a population with limited treatment options, FIND-CKD could significantly expand the therapeutic role of finerenone in kidney care and improve outcomes for patients without diabetes [71]. The study is estimated to be completed in early 2026.

### 5.3. Aldosterone Synthase Inhibitors

Inhibiting aldosterone at its source offers a novel therapeutic approach for reducing the renal and cardiovascular complications associated with CKD. Unlike MRAs, which block aldosterone at its receptor, aldosterone synthase inhibitors, ASIs, act upstream by suppressing aldosterone production through the selective inhibition of aldosterone synthase (CYP11B2). This approach has the potential to suppress both genomic MR-mediated effects and non-genomic, MR-independent actions of aldosterone, which contribute to inflammation and fibrosis. These non-genomic pathways may even be exacerbated by MRAs, which elevate circulating aldosterone levels as a compensatory response, though the clinical relevance of this is still under debate [59]. ASI treatment preserves MR function so that other MR ligands, e.g., glucocorticoids, can maintain a basal mineralocorticoid activity. Early efforts to develop ASIs were hindered by the challenge of achieving selectivity for CYP11B2 without interfering with cortisol synthesis (via CYP11B1), as seen with first-generation agents like osilodrostat (LC1699). However, newer ASIs such as baxdrostat, lorundrostat, vicadrostat, and dexfadrostat have demonstrated improved specificity and tolerability [94]. While the majority of ASIs mainly target uncontrolled/resistant hypertension or primary aldosteronism, vicadrostat is in development for people with CKD with the intention to slow the decline in kidney function and to reduce the risk of cardiovascular events. A phase 1 study evaluated the safety and early efficacy of vicadrostat in diabetic patients with albuminuric CKD and showed a significant decrease in UACR in comparison with placebo, while in a recent phase 2 trial, vicadrostat alone or in combination with empagliflozin significantly reduced UACR in patients with CKD already receiving optimized RAS inhibition, without impacting cortisol levels. Despite some dropout due to dose-related adverse events, vicadrostat showed a dose-dependent reduction of albuminuria, and when used on top of empagliflozin, exhibited additive efficacy [95]. The EASi-KIDNEY trial, a phase 3 trial designed to evaluate whether vicadrostat, given on top of standard of care including empagliflozin, will reduce the risk of kidney disease progression, hospitalization for heart failure, or death from cardiovascular disease in people with CKD, is currently recruiting and study completion is estimated to be in mid-2028 [96]. A phase 3 study to investigate the efficacy and safety of baxdrostat in combination with dapagliflozin on CKD progression in participants with CKD and high blood pressure is also ongoing and is estimated to be completed at the end of 2027 [97].

## 6. Conclusions and Future Perspectives

In physiological conditions, aldosterone plays a pivotal role in maintaining blood pressure and fluid balance. Chronically elevated aldosterone levels, however, contribute to the development of CKD by promoting fibrosis through proinflammatory and oxidative mechanisms. While MRAs have proven effective in mitigating these harmful effects, the clinical use of steroidal MRAs remains limited due to well-known side effects. In response, novel non-steroidal MRAs such as finerenone have demonstrated efficacy in slowing CKD progression and reducing residual cardiorenal risk, with significantly fewer side effects. Nevertheless, since aldosterone also exerts non-genomic, non-MR-mediated effects, ASIs have emerged as a promising strategy to block both the genomic and non-genomic actions of aldosterone. Current findings support the continued investigation of aldosterone synthase inhibition as a promising and mechanistically distinct approach to slowing CKD progression and reducing residual cardiorenal risk.

## Data Availability

No new data were created or analyzed in this study. Data sharing is not applicable to this article.

## Abbreviations

The following abbreviations are used in this manuscript:

11β-HSD2	11β-hydroxysteroid dehydrogenase type 2
ACEi	angiotensin converting enzyme inhibitor
Ang II	angiotensin II
ARB	angiotensin receptor blocker
ASI	aldosterone synthase inhibitor
CKD	chronic kidney disease
CYP11β2	cytochrome P450 11β2, aldosterone synthase
eGFR	estimated glomerular filtration rate
ENaC	epithelial sodium channels
ESKD	end-stage kidney disease
Gal-3	galectin-3
HFrEF	heart failure with reduced ejection fraction
MR	mineralocorticoid receptor
MRA	mineralocorticoid receptor antagonist
NGAL	Neutrophil Gelatinase-Associated Lipocalin
NT-proBNP	N-terminal pro B-type natriuretic peptide
PAI-1	plasminogen activator inhibitor-1
RAAS	renin–angiotensin–aldosterone system
Rac-1	Ras-related C3 botulinum toxin substrate 1
RAS	renin–angiotensin system
ROS	reactive oxygen species
SGLT2	sodium–glucose transport protein 2
TGF-β	transforming growth factor-beta
UACR	urinary albumin-to-creatinine ratio
